# Recent Discoveries of Diagnostic, Prognostic and Predictive Biomarkers for Pancreatic Cancer

**DOI:** 10.3390/cancers12113234

**Published:** 2020-11-02

**Authors:** Andrii Khomiak, Marius Brunner, Maximilian Kordes, Stina Lindblad, Rainer Christoph Miksch, Daniel Öhlund, Ivonne Regel

**Affiliations:** 1 Shalimov National Institute of Surgery and Transplantology, 03058 Kyiv, Ukraine; kh.ndrew@gmail.com; 2 Department of Gastroenterology, Endocrinology and Gastrointestinal Oncology, University Medical Center, 37075 Goettingen, Germany; marius.brunner@med.uni-goettingen.de; 3 Department of Upper Abdominal Diseases, Karolinska University Hospital, 14186 Stockholm, Sweden; maximilian.kordes@ki.se; 4 Department of Clinical Science, Intervention and Technology (CLINTEC), Karolinska Institutet, 17177 Stockholm, Sweden; 5 Department of Radiation Sciences, Sweden and Wallenberg Centre for Molecular Medicine, Umeå University, 90187 Umeå, Sweden; stina.lindblad@umu.se; 6 Department of General, Visceral and Transplantation Surgery, University Hospital, LMU Munich, 81377 Munich, Germany; rainer.miksch@med.uni-muenchen.de; 7 Department of Medicine II, University Hospital, LMU Munich, 81377 Munich, Germany

**Keywords:** pancreatic ductal adenocarcinoma, pancreatic cancer, protein biomarkers, microbiome biomarkers, metabolome biomarkers, DNA biomarkers, immune biomarkers

## Abstract

**Simple Summary:**

Biomarkers for cancer diagnosis, prognosis and prediction are important tools and an urgent need in precision medicine for pancreatic cancer. In recent years, many experimental and clinical studies aimed at identifying new biomarkers for pancreatic ductal adenocarcinoma. In the review, we summarized current investigations on using novel protein markers, cell-free DNA, metabolome compounds, immune and stroma signatures and microbiome compositions as biomarkers for pancreatic cancer. Our comprehensive overview shows that although there are new promising biomarkers, CA 19-9 remains currently the only regularly used and validated biomarker for pancreatic cancer in clinical routine.

**Abstract:**

Pancreatic ductal adenocarcinoma (PDAC) is an aggressive disease with a dismal prognosis that is frequently diagnosed at an advanced stage. Although less common than other malignant diseases, it currently ranks as the fourth most common cause of cancer-related death in the European Union with a five-year survival rate of below 9%. Surgical resection, followed by adjuvant chemotherapy, remains the only potentially curative treatment but only a minority of patients is diagnosed with locally resectable, non-metastatic disease. Patients with advanced disease are treated with chemotherapy but high rates of treatment resistance and unfavorable side-effect profiles of some of the used regimens remain major challenges. Biomarkers reflect pathophysiological or physiological processes linked to a disease and can be used as diagnostic, prognostic and predictive tools. Thus, accurate biomarkers can allow for better patient stratification and guide therapy choices. Currently, the only broadly used biomarker for PDAC, CA 19-9, has multiple limitations and the need for novel biomarkers is urgent. In this review, we highlight the current situation, recent discoveries and developments in the field of biomarkers of PDAC and their potential clinical applications.

## 1. Introduction

Across patients with different types of malignant tumors, the stage at diagnosis, prognosis and success rates of anti-cancer treatment vary widely [1]. These disparities correspond to the complex nature of cellular, inter-cellular and organ-specific mechanisms that drive tumorigenesis. Biomarkers reflecting changes on either of these levels can be objectively measured to detect malignant and other diseases, to monitor their course and to select treatment strategies [2]. Pancreatic ductal adenocarcinoma (PDAC) is a highly malignant tumor of the exocrine pancreas with a poor prognosis. Although less common than other malignant diseases, it currently ranks as the fourth most common cause of cancer-related death in the European Union [1]. In four out of five cases, PDAC is diagnosed in a locally advanced or metastatic stage when it is not amenable to surgery [3]. The five-year survival rate among patients diagnosed with PDAC is below 9% [4,5]. Biomarkers can improve the care of cancer patients essentially in three different ways. Diagnostic markers can help to detect PDAC at an earlier and potentially curable stage. Prognostic markers can provide valuable information to patients and assist clinicians to adjust their treatment strategies according to the aggressiveness of the disease. Predictive markers can help to foresee how an individual patient might respond to a treatment and different treatment protocols can be selected based on a biological rationale. Because biomarkers reflect pathophysiological or physiological processes inherently linked to the disease there might also be a substantial overlap between the diagnostic, prognostic and predictive capacities of some markers. This review will focus on the current situation in the field of biomarkers of PDAC, highlight recent discoveries and discuss their potential in diagnostic, prognostic and predictive applications.

## 2. Clinical and Laboratory Features as Biomarkers

The term biomarker usually denotes a molecular marker, but even clinical parameters or other clinicopathological features might be relevant for the diagnosis, prognosis or prediction of treatment response. Several prognostic scores based on clinical features and routine laboratory values have been validated for PDAC [6]. The Glasgow Prognostic Score (GPS) incorporates serum C-reactive protein and albumin levels and has been shown to be a reliable independent prognostic factor in pancreatic, colorectal, gastro-esophageal, hepatocellular and other malignant tumors [7]. Moreover, the neutrophil/lymphocyte ratio (NLR), platelet/lymphocyte ratio (PLR), the Prognostic Index (combination of C-reactive protein and white cell count) and Onodera’s Prognostic Nutritional Index have also been shown to be associated with cancer specific survival in patients with various cancers, including PDAC [8,9,10]. Using clinical data, such as age, tumor stage, alanine aminotransferase and albumin levels, several nomograms have been generated and validated to predict survival of patients with PDAC [11,12,13,14,15]. Notably, some of these nomograms have a global scope and can predict survival across all stages of PDAC [14], while others focus on specific stages, e.g., patients with locally advanced PDAC and aim to improve pre-operative treatment [15]. For guidance of chemotherapy regimens several nomograms have been developed. Nomograms are used to assess the efficacy of gemcitabine monotherapy, nab-paclitaxel/gemcitabine or FOLFIRINOX, which are the most widely used regimens for advanced PDAC [12,13,16]. In summary, GPS, NLR, PLR and several nomograms could discriminate patient sub-groups with different survival outcomes or guide therapy choices, but several issues, such as ill-defined cut-off values or a lack of validation, limit their use in clinical practice at the time.

## 3. Protein Biomarkers

To date, CA 19-9 is the only broadly accepted biomarker of PDAC. CA 19-9 is a sialyl-Lewis A tetrasaccheride, which about 10% of the population with a Lewis-negative genotype cannot express [5]. It has been extensively studied since the 1980s and it is currently the only diagnostic marker for PDAC approved by the U.S. Food and Drug Administration (FDA). In a meta-analysis including 26 studies with more than 2200 patients, the median sensitivity of elevated CA 19-9 to detect PDAC was 79% and the median specificity was 82% [17]. However, it is important to note that other malignant conditions, such as colorectal, lung and liver cancer or even benign diseases, such as cholestasis, pancreatitis or systemic lupus erythematosus, may cause increased CA 19-9 levels [5]. In one of the largest multicenter datasets of more than 1900 pancreatic cancer patients, it was shown that the classification of CA19-9 levels has a prognostic value. Normal compared to increased CA 19-9 levels were associated with a hazard ratio (HR) for death between 0.68 (advanced stages III–IV) and 0.77 (stage I–II). Thus, high CA 19-9 levels have been implicated as an unfavorable prognostic marker that is linked to a short overall survival in patients with PDAC [18,19]. An important limitation of many studies investigating the prognostic value of CA 19-9 is their retrospective design and heterogeneous patient populations, which make it difficult to gauge the extent to which differences in survival are associated with different CA 19-9 levels. Notably, analyses of some prospective clinical trials have shown an association of CA 19-9 with survival in some cases, while there has not been any significant association in other trials. Therefore, some investigations postulate that CA 19-9 kinetics rather than baseline level correlate with overall survival [20]. A potential explanation for these disparate observations is the substantial variations of threshold values of CA 19-9 across different studies and that there is no universally accepted definition of a decrease of CA 19-9 as a proxy of tumor response. Thus, CA 19-9 needs to be interpreted with caution and in the clinical context of individual cases. Nevertheless, there is a large body of evidence that the CA 19-9 response to chemotherapy correlates with treatment outcomes and overall survival [21].

In addition to CA 19-9, the diagnostic or prognostic utility of several other carbohydrate antigens has been tested. Carcinoembryonic antigen (CEA) is widely used as a marker in different tumor entities and increased levels of CEA have been reported in more than 60% of PDAC patients [22]. Although the combination of CEA with CA 19-9 might improve the specificity of a diagnostic PDAC test, the sensitivity was shown to be poorer than for CA 19-9 alone [23]. CA 125 is an antigenic determinant on a high–molecular-weight glycoprotein recognized by a monoclonal antibody (OC 125), which was originally raised against an epithelial ovarian carcinoma cell line. CA 125 is a tumor marker in different types of cancers, such as ovarian, breast and lung neoplasms [24]. Its utility was also confirmed for the diagnosis of PDAC with slightly less sensitivity than CA 19-9 [25,26]. When used in combination with CA 19-9, CA 125 resulted in an improved specificity and could even yield clinically meaningful sensitivity for previously undiagnosed cases of PDAC [23,27]. Other carbohydrate markers, such as CA 50, CA 72-4 and CA 242, have been extensively studied in PDAC patients [28,29,30,31]. Although they exhibited less sensitivity than CA 19-9 for the diagnosis of PDAC, some studies reported improved specificity (Table 1). A large variety of combinations of carbohydrate markers was proposed to improve both specificity and sensitivity of the test [28,30,32]. Still, neither single carbohydrate marker tests, nor a combination of carbohydrate antigens are currently used in clinical practice, possibly due to a lack of standardization and absence of validation on larger cohorts.

In addition to carbohydrate antigens, several other proteins have been studied as PDAC serum markers. The macrophage inhibitory cytokine 1 (MIC-1) is a distant member of the transforming growth factor β superfamily and was originally found in colorectal, breast and prostate cancer [39,40,41]. Koopmann et al. measured serum MIC-1 levels of 80 patients with PDAC, 30 patients with ampullary cancer or cholangiocarcinoma, 42 patients with benign pancreatic tumors, 76 patients with chronic pancreatitis (CP) and 97 healthy subjects [42]. Serum MIC-1 levels were significantly higher in patients with PDAC and in patients with ampullary cancer or cholangiocarcinoma than in patients with benign pancreatic neoplasms, CP or in healthy controls. These differences were comparable to the CA 19-9 levels across groups [42]. Moreover, MIC-1 could differentiate patients with early-stage resectable PDAC significantly better from healthy individuals than CA 19-9 [43]. Moreover, Mucin glycoproteins, among them Mucin 1 (MUC1), are highly expressed in pancreatic cancer and detectable with monoclonal antibodies, such as PAM4 (clivatuzumab), that are reactive to MUC1 [44]. Using pancreatic cancer and pancreatitis specimens, the overall sensitivity and specificity of PAM4 to detect PDAC were 77% and 95%, respectively, with a Receiver Operating Characteristic (ROC) area under the curve (AUC) of 0.89. In this study, the accuracy of the PAM4 immunoassay assay was significantly better than that of CA19-9 [45].

PDAC is characterized by a dense fibrotic stroma that surrounds the cancer cells. During tumor progression, the collagen-rich stroma is modulated and rearranged to support the expansion of cancer cells. This is a dynamic process orchestrated by cancer cells and cancer-associated fibroblasts (CAFs), where fragments from collagens and other extracellular matrix (ECM) components of the stroma are spread to the circulation [46,47,48]. Thus, Osteopontin (OPN) is a protein of the ECM, with a variety of functions in cell adhesion and migration, inflammatory reaction and apoptosis. OPN has been found to be upregulated in PDAC and was linked to invasiveness as well as metastatic growth of pancreatic cancer cells [49,50]. Elevated OPN discriminated pancreatic cancer patients from healthy individuals with a sensitivity of 80% and a specificity of 97%, but did not perform significantly different from CA 19-9 [51]. Another study confirmed that OPN could distinguish PDAC from CP and healthy controls, but ROC analysis showed that OPN failed to improve diagnostic capability over CA 19-9 with an AUC of 0.72 and 0.92, respectively [52]. Another prognostic biomarker of PDAC was uncovered in a recent study that demonstrated an association of specific collagen fragments with the outcome of PDAC patients. Matrix metalloprotease (MMP)-degraded type I collagen (C1M), type III collagen (C3M), type IV collagen (C4M) and a pro-peptide of type III collagen (PRO-C3) were measured in the serum of patients prior to administration of 5-fluorouracil-based therapy for advanced-staged PDAC. High levels of circulating collagen fragments were consistently associated with a shorter overall survival [53].

In addition to blood-circulating biomarkers, the expression of specific proteins in tumor tissue is associated with the prognosis of PDAC or response to specific therapies. The human equilibrative nucleoside transporter 1 (hENT1) is a predictive tissue marker for the response to gemcitabine treatment [54,55]. A systematic analysis of the association of high versus low hENT1 expression with overall survival in gemcitabine-treated patients revealed hazard ratios (HR) for death in the range of 0.26 to 0.51 [56]. Another clinically interesting protein is dihydropyrimidine dehydrogenase (DPD). This enzyme is involved in the catalyzation of the chemotherapeutic drug 5-fluorouracil (5-FU) used in several treatment regimens for pancreatic cancer [57,58]. The correlation between treatment response and the presence of hENT1 and DPD in pancreatic tumor cells has been studied in the population from the ESPAC-4 trial. They were able to correlate high levels of DPD to worse prognosis for patients treated with 5-FU and established it as a negative predictive factor [59]. Moreover, low expression of the leucine-rich repeat-containing G-protein-coupled receptor (LGR5) was previously associated with a shorter survival in gastrointestinal cancer [60,61]. LGR5 is expressed in the cytoplasm of PDAC cells and in the basolateral membrane of a subset of endocrine cells of the human pancreas [62]. Among 78 PDAC cases, the expression of LGR5 was significantly higher in low histological grade (G1–G2) and early clinical stage. Congruently, the median overall survival (OS) of patients with tumors with a high LGR5 expression was 36 months compared to a median OS of 17 months of patients with a low LGR5 expression [63]. Overall, CA19-9 remains the only reliable, validated marker for prognostic use in patients with PDAC despite several tumor and serum proteins with a similar efficacy that could be used as biomarkers in the future. Particularly promising are the combinations of different markers. Still, further studies are required to validate novel protein biomarkers and implement them in clinical practice.

## 4. DNA Biomarkers

Cell free DNA (cfDNA) consists of circulating double-stranded DNA molecules that can be found in blood plasma or serum. Although the origin of cfDNA is still under debate, there is evidence that DNA is released during normal cell metabolism or apoptosis or necrosis. While cfDNA can be also found in healthy individuals, cancer patients have a significant higher concentration of cfDNA in the blood [64]. However, a direct quantification measurement of the cfDNA in plasma or serum was unsuccessful for the diagnosis of cancer [65]. Still, detection of tumor-specific mutations and epigenetic alteration in cfDNA is a promising direction for diagnostic and prognostic “liquid biopsies” in patients with different kind of tumors, including PDAC [66]. Pancreatic cancer was one of the first malignant diseases, in which specific single nucleotide variants (SNV) mutations in the *KRAS* (Kirsten rat sarcoma viral oncogene homolog) gene were detected in cfDNA [67]. Mutations in *KRAS* stand out among the genetic aberrations of PDAC, because they are present in more than 85% of the cancer cases and the vast majority of these mutations cluster into only three different variants in exon 12; G12D, G12V and G12R [68,69,70]. *KRAS* mutations (*KRAS*^mut^) occur early in PDAC development [71]. Their high conservation and frequency make them a genetic hallmark of PDAC, and thus, *KRAS*^mut^ a potential genomic biomarker. Tumor-derived cfDNA in the plasma of PDAC patients are a small fraction among all cfDNA derived from healthy cells with fragments mainly in the 185–200 bp range [72]. While *KRAS* mutations occur in the majority of PDAC, the more complex mutational changes affecting other genes and the spatial heterogeneity of PDAC can also be identified in cfDNA [73,74,75,76,77]. Similarly, copy number alterations of PDAC have been detected in cfDNA [78]. Unsurprisingly, cfDNA and especially mutant *KRAS*, has been suggested as a diagnostic tool for PDAC and there is a high concordance between SNV in cfDNA and mutations in matched tissue samples [79]. In a large retrospective dataset of 403 patients with resectable PDAC and healthy individuals, the sensitivity of KRAS mutations in cfDNA was found to be 30% with 1/182 false positive samples [80]. Similar findings were observed in another cohort of 437 patients with PDAC, 141 patients with CP and 394 healthy individuals, where the *KRAS*^mut^ detection frequency was 21.1% among pancreatic cancer patients and 4.3% and 3.7% in pancreatitis patients and healthy individuals, respectively [81]. The discrimination between non-malignant and pre-malignant pancreatic lesions, such as intraductal papillary mucinous neoplasia (IPMN), and manifested tumors was improved by combining *KRAS* and *GNAS* mutation analysis as the assay performed quite well at discriminating between IPMN and PDAC [82]. In the CancerSEEK project, the sensitivity of cfDNA analysis was improved up to 75% by the incorporation of protein markers with few limitations in early detection of localized diseases [83]. At least one further study supports the incorporation of the protein markers CA19-9 and THBS2 into cfDNA analysis [84]. Another approach to overcome the limitations of *KRAS*^mut^ alone might include the analysis of degraded DNA fragments [85]. In addition, by analyzing the methylation status of two genes, *ADAMTS1* and *BNC1*, in cfDNA, a 94.8% sensitivity and 91.6% specificity was achieved, supporting other reports that methylation patterns of cfDNA are able to identify early stage cancer [86,87]. Nevertheless, for patients with advanced PDAC or patients undergoing tumor surgery, increased plasma levels of tumor-associated cfDNA have been correlated to poorer clinical outcomes across several studies (Table 2). The majority of these studies focused on the detection of *KRAS* codon 12 mutations and used digital PCR and occasionally other methods such as peptide-nucleic acid clamp PCR and panel sequencing [88,89,90]. The detection rate of *KRAS*^mut^ in cfDNA varies across studies in the range of 26–42% [82,91,92].

In a small series of patients undergoing chemotherapy, the level of *KRAS*^mut^ in cfDNA correlated also with the radiological tumor response or disease progression [94]. In a cohort of patients, who underwent resection of a localized PDAC, the presence of *KRAS*^mut^ cfDNA was detected in the plasma of 22/51 patients (43%) at the time of diagnosis. Using cfDNA to follow disease progression, *KRAS* mutations were detected as early as 3.1 months after surgery, compared to 9.6 months using standard CT. An early relapse of *KRAS*^mut^ in cfDNA predicted a clinical relapse and was associated with poor survival [103].

DNA hypermethylation of promoter regions is a well-known mechanism for the inactivation of tumor suppressor genes associated with carcinogenesis [104]. It has been previously shown that DNA hypermethylation has a prognostic value and may be used as an independent predictor of survival in different types of cancer [105,106,107]. Henriksen et al. examined the correlation between hypermethylated genes in plasma-derived cfDNA and survival of patients with PDAC [108]. Methylation-specific PCR of 28 genes was performed in a cohort of 95 patients with pancreatic cancer. Patients with more than 10 hypermethylated genes were found to have a HR for death of 2.03 (95% CI; 1.15–3.57) compared to patients with fewer hypermethylated genes. The authors have designed three prediction models based on cfDNA hypermethylation, which stratify PDAC patients into risk groups according to survival and have the potential to provide additional information as prognostic biomarkers [108]. Methylation profiling analysis in leukocyte DNA has also been shown to discriminate cancer patients from non-cancer controls, an approach that was pioneered in small-cell lung cancer [109]. Measuring methylation levels at 1505 CpG sites in treatment-naive leukocyte DNA from 132 PDAC patients and 60 healthy controls, Pedersen et al. could derive a prediction model that consisted of five CpG sites (IL10_P348, LCN2_P86, ZAP70_P220, AIM2_P624 and TAL1_P817) to discriminate PDAC patients from controls. Moreover, one CpG site alone, LCN2_P86, could differentiate patients with localized, resectable tumors from healthy individuals [110]. These data suggest that most of the early PDAC cases could be diagnosed non-invasively using diagnostic tests based on a few specific genetic alterations. Prognostic and predictive models based on liquid biopsies may also allow for better stratification of patients according to survival and tailor future therapy regimens.

## 5. Metabolomic Biomarkers

Analyzing the metabolome is a novel approach for the detection of cancer signatures, although there are currently no routinely used metabolic markers in clinical practice for diagnosis, prognosis assessment or prediction of the response to chemotherapy. There is, however, an increasing number of studies that show differences between metabolic profiles of PDAC patients, patients with other conditions or healthy controls [111,112,113,114]. In a case-control study, Mayerle et al. investigated the metabolic profile of blood samples from 914 patients with PDAC, CP, liver cirrhosis, healthy and non-pancreatic disease controls. The authors identified a biomarker signature (nine metabolites and additionally CA 19-9) that reliably distinguished PDAC from CP with a negative predictive value for cancer of 99.9% (95% CI 99.7–99.9%) [112]. In another study of 106 patients who underwent surgery for PDAC, tissue specimens were analyzed to find metabolic biomarkers associated with long-term survival, using metabolomics analysis. Network analysis revealed higher levels of glucose, ascorbate and taurine to be correlated with long-term survivorship. At the same time, long-time survivors tended to have decreased levels of choline, ethanolamine, glycerophosphocholine, phenylalanine, tyrosine, aspartate, threonine, succinate, glycerol, lactate, glycine, glutamate, glutamine and creatine. Among these metabolites, the level of ethanolamine had the highest accuracy to distinguishing long-term from short-term survivors. The AUC was 0.86  ±  0.1, with a sensitivity and specificity of 77.8% and 75%, respectively [115]. Another study analyzed the metabolomic profiles of tumor tissues from 25 patients, who underwent curative resection and adjuvant gemcitabine-based therapy. Elevated lactic acid levels were found in the tumors of patients with poor clinical outcomes after gemcitabine treatment. In contrast, patients with low levels of lactic acid and higher protein expression of hENT1 were found to have a significantly longer survival time than all other groups [116]. A general weakness with metabolomic studies, especially retrospective ones, is that there are many aspects that need to be considered when comparing the results. The collected sample material, the sample preparation, the analytical platform and interpretations can influence the outcome. There is a need for further standardizations and validation of candidate metabolic signatures, preferably through studies with a robust design [117]. Although there is a need for further standardizations and validation of candidate metabolic signatures, metabolomics is a promising approach to provide new useful diagnostic, prognostic or predictive markers for PDAC patients.

## 6. Immune and Stroma Biomarkers

Pancreatic cancer is traditionally considered immune-cold neoplasm employing a great number of mechanisms for immune evasion. It possesses a complex tumor microenvironment (TME), which enables PDAC to evade the host immune system and explains low effectiveness of chemo-, radio- and novel immunotherapeutic approaches [118,119]. As been already shown in a variety of cancer types, a better understanding of the immune landscape could provide new rational targets for therapy and allow for better prognostic stratification than conventional staging systems [120,121,122,123,124].

**Figure 1:**
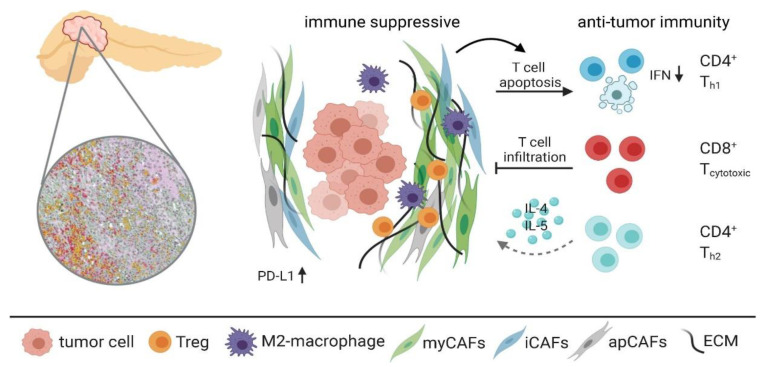
Immune infiltrates of pancreatic ductal adenocarcinoma form an immune suppressive environment. Anti-tumor immunity is suppressed through T-cell apoptosis, inhibition of cytotoxic T-cell recruitment and reduced secretion of pro-inflammatory cytokines, such as IL-4 and IL-5. PD-L1: Programmed death-ligand 1, Treg: regulatory T-cell, myCAFs: myofibroblastic cancer-associated fibroblast (CAF), iCAFs: inflammatory CAFs, apCAFs: antigen-presenting CAFs. Created with BioRender.com.

The tumor microenvironment (TME) of pancreatic cancer consists of stromal and cellular compartments, which are both linked to prognosis and survival of patients. PDAC has a unique and abundant stromal component, comprising up to 90% of the tumor volume. While cellular infiltrates were originally considered to play a primary role in TME of PDAC, it has been recently proved that the stromal compartment is not a silent bystander. Pancreatic stellate cells (PSC) are considered primarily responsible for the synthesis of desmoplastic stroma. Their interaction with PDAC cells and effects on tumor microcirculation support tumor growth, metastatic spread and chemoresistance [124,125,126,127,128]. Several subtypes of pancreatic stroma were identified and associated with patients’ prognosis. Erkan et al. previously introduced the activated stroma index [129]. Furthermore, Knudsen et al. investigated in their study a cohort of 109 PDAC cases using histologic analysis and immunostaining to evaluate stromal characteristics of the tumor. The study identified three distinct stromal phenotypes based on the number of PSCs, presence of mature collagen fibers and loose extracellular matrix: immature, intermediate and mature. These phenotypes were significantly associated with survival times with immature subtype yielding shorter OS [130]. In the study of Moffitt et al., they utilized nonnegative matrix factorization to identify two different prognostic stromal subtypes, which were defined as “active” and “normal.” The active stroma subtype was correlated with a worse median survival of 15 month and a one-year survival rate of 60%, while patients with a normal stroma had a median survival time of 24 months and a one-year survival rate of 82% [131]. Deactivation of the tumor stroma has a significant impact on PDAC biology and therapeutic resistance [121]. Modulating the stroma in PDAC may be a tool to improve the effect of therapy. Neesse et al. could show that SPARC ablation reduces the collagen-rich microenvironment in murine models [132]. Furthermore, therapeutic treatments targeting the stroma aim to change its composition and decrease chemoresistance in PDAC patients. In a study by Gorchs et al., the authors showed that vitamin D3 analogues, that inhibit CAF proliferation and migration, appear to have dual functions in the context that calcipotriol reduces the tumor supportive activity of CAFs but at the same time reduces T cell effector functions, which could have important clinical implications [133]. In a study by Olive et al., an inhibitor of the hedgehog signaling pathway was shown to deplete the fibrotic stroma, and enhance chemotherapy delivery to cancer cells in a mouse model of PDAC [134], but these findings could not be repeated in a human setting. In contrary, follow up studies have shown that inhibition of the hedgehog pathway [135], or depletion of alpha-smooth muscle actin expressing CAFs [136], both leads to a depletion of the tumor stroma, but also to a more aggressive disease and reduced survival in mouse models of PDAC. This highlights the complexity of the tumor stroma, in particular for CAFs, and indicates that some stromal elements within the stroma instead restrain tumor progression. Recently, is has become clear that functionally distinct subtypes of CAFs are present in the PDAC stroma, such as myofibroblastic CAFs (myCAFs), inflammatory CAFs (iCAFs) [137] and antigen presenting CAFs (apCAFs) [138], all with potential different roles in the pathophysiology of the disease (Figure 1). Altogether, this indicates that a future therapeutic strategy aiming at depleting the tumor stroma and/or CAFs has to target specific components of the stroma, and not the stroma in general, since this also might eliminate elements with tumor restraining functions. This also shows the great need of biomarkers that correctly can reflect the balance between the tumor restraining and tumor promoting elements of the stroma.

Tumor-infiltrating immune cells are considered to be one of the most important factors in the TME defining biological behavior of the tumor [139,140,141]. Multiple studies investigated intratumoral immune cell composition using immunohistochemistry and revealed an association with prognosis and survival of PDAC patients. The study by Tewari et al. has found a correlation between the presence of CD3 and CD8 positive lymphocytes in the tumor and the tumor differentiation grade. A higher presence of intratumoral and stromal CD3 and CD8 stained lymphocytes was associated with a lower tumor grade and better survival [142]. In a study by Ino et al., shorter survival was observed in the presence of high levels of tumor-infiltrating CD163+ or CD204+ M2 macrophages, neutrophils or a high density of regulatory T-cells (FoxP3+ cells). The same study found a higher level of tumor-infiltrating CD4+ T-cells, CD8+ T-cells or the ratio of HLA-DR+ and CD68+ M1 macrophages to pan-macrophages to convey a better prognosis and longer survival times for the patient [143]. Another study by Miksch et al. confirmed the importance of intra- and peritumoral immune cell infiltrates for patients’ prognosis and survival. The study found that high infiltration of CD3+, CD8+ and CD20+, which were predominantly observed in peritumoral “hot spots”, correlated significantly to OS and progression-free survival (PFS) in PDAC patients. A little number of immune cell infiltrates were found intratumorally, confirming a superb ability of PDAC to evade the host immune system. Importantly, the study did not find any association of patient survival times with the distance of the mentioned immune cell “hot spots” and tumor front [144]. Programmed death-1 ligand (PD-L1) has been suggested to play a crucial role in this immune system escape through negative regulation of T-cell function, since higher PD-L1 levels were inversely correlated with the quantity of tumor immune cell infiltrates [145]. Most attempts to apply immunotherapy regimens for the treatment of PDAC proved to be ineffective. Immunosuppressive tumor milieu and dense fibrotic stroma modulated by CAFs are thought to be responsible for the failure of most approaches (Figure 1). There are, however, a few promising trials published. Adaptive T-cell transfer, pancreatic cancer vaccines, immune checkpoint inhibitors and co-stimulatory agents are the approaches currently under investigation for the treatment of pancreatic cancer [146,147,148]. Adaptive T-cell transfer is an approach with ex-vivo activation of host T-cells with subsequent reinfusion. Promising results were published on this strategy that was applied in hematologic malignancies, but efficacy of the approach for the treatment of solid neoplasms is yet to be determined [149,150,151]. Of high interest are results of a phase I trial, in which Beatty et al. investigated safety and efficacy of adoptive cell therapy with autologous mesothelin-specific chimeric antigen receptor T-cells in six patients with chemotherapy-refractory metastatic PDAC. Stable disease with progression-free survival times of 3.8 and 5.4 months was achieved in two patients. Total tumor metabolic active volume (MAV) on positron emission tomography was stable in three patients and decreased in one patient, who also had decreased activity of metastatic lesion in the liver [146]. Pancreatic cancer vaccines have gained a lot of attention recently. Mutated RAS peptide vaccine was found to be of clinical benefit in adjuvant setting [152,153]. In the study of Wedén et al. the rate of immune response is reported to be as high as 85% with 10-year survival of 20% in responders versus zero in a cohort of non-vaccinated patients [152]. Another study reports a peptide-specific immunity induced in 58% patients with median survival of 148 days vs. 61 days, in vaccinated and non-vaccinated patients, respectively [153]. Granulocyte-macrophage colony-stimulating factor-secreting tumor vaccine was found to be effective in adjuvant settings, when combined with chemoradiation, gemcitabine or Listeria vaccine that expresses mesothelin [154,155,156]. The latter combination of two vaccines demonstrated an improved OS of 6.1 vs. 3.9 months compared to single vaccine alone and was even granted FDA breakthrough designation [156]. Groeneveldt et al. highlighted the importance of preconditioning in PDAC. They showed results sensitizing the tumor microenvironment for subsequent CD3-bsAb therapy in combination with an oncolytic reovirus [157]. A limited number of studies have been completed on immune checkpoint inhibitors with disappointing results published both on CTLA-4 and PD-L1 inhibitors [147,148]. However, it has been hypothesized that PDAC with deficiency in DNA mismatch repair (MMR) has increased sensitivity to immune checkpoint blockade with anti-PD-1 antibodies. A recent study of Le et al. investigated the effects of PD-1 blockade using pembrolizumab in MMR-deficient tumors independently of the tissue of origin and included 86 patients with 12 different tumor types (including PDAC). Fifty-three percent of patients achieved radiological response, whereby a complete response was observed in 21% of patients. Response was durable with median progression-free and overall survival still not reached [152]. Rapid expansion of neoantigen-specific T-cell clones that were reactive to mutant neopeptides, created by the multiple somatic mutations in cancers, were found in the tumor of responders. These data support the hypothesis that a large proportion of mutant neoantigens in MMR-deficient pancreatic cancer makes the tumor susceptible to immune checkpoint blockade [158].

In summary, stromal subtypes influence, together with inflammatory cytokines levels, the prognosis of the patients with PDAC. Stromal maturity as well as activation status are considered to be important prognostic features. A general weakness of studies investigating immune markers for prognostic or predictive implications is a retrospective design limiting their future clinical application. A very low number of studies show promising results for immunotherapy approaches in PDAC, in which a small subset of PDAC patients with MMR deficiency might benefit. These data show that the immune milieu of PDAC is very complex and requires combinatorial approaches for prognostic and predictive application and increasing efforts to develop future immunotherapy regimens. A better understanding of the mechanisms that regulate the PDAC immunosuppressive environment could reveal specific disease subtypes with enhanced prognostic significance and allow for additional targeted treatment approaches.

## 7. Microbiome Biomarkers

The correlation between the gut and oral microbiome and pancreatic cancer has become the center of attention recently and is discussed in detail in an excellent review from Picardo et al. [159]. Hence, many recent studies have focused on the tumor microbiome to discover prognostic factors for PDAC. If understood better, microbiome screening methods might be able to predict the risk of pancreatic cancer and, in the course of the disease, help to decide the best treatment options. The tumor microbiome in pancreatic cancer is strongly linked to both the oral and gut microbiome. Immunofluorescent-labeled bacteria given orally to mice were found in the tumor six to eight weeks later, maybe even providing a potential possibility to carry therapeutic agents into the tumor tissue [158,160,161]. Prognostic relevance of the tumor microbiome was shown by Mitsuhashi et al., who demonstrated that tumors harboring fusobacterium species, which occurred in approximately 10% of cases, have a significantly poorer prognosis (HR 2.16, 95%-CI, 1.12–3.91), underlining a possible prognostic application after acquiring biopsies or surgery specimen [162]. However, other studies have identified fusobacteria to be associated with a reduced PDAC risk, postulating that there might be a different role between oral fusobacteria and tumor fusobacteria [163]. A correlation between the gut microbiome and the response to immunotherapy has been shown in other malignancies [164,165]. In a recent publication, Riquelme et al. showed that the gut microbiome of long-term pancreatic cancer survivors was significantly more diverse than that of short-term survivors [166,167,168]. Most strikingly, tumor-bearing mice, who underwent the transplantation of both oral and gut microbiomes, were found to survive significantly longer with the fecal transplant of long-term survivors, postulating a strong interaction between tumor biology and gut microbiome. Furthermore, Mendez et al. described that pancreatic cancer patients show a significantly elevated serum polyamine level as a consequence of microbial dysbiosis, which is known to be actively assimilated by the host and eventually utilized by rapidly dividing cells for proliferation being of importance in the process of tumorigenesis [169]. The response to chemotherapy could also be linked to the tumor microbiome through intra-tumoral bacteria, which might contribute to drug resistance of PDAC. Of 113 human pancreatic cancer tissue samples that were tested, 86 (76%) were positive for bacteria, mainly Gammaproteobacteria, which was previously correlated to gemcitabine-resistance [170].

In summary, the data show that the tumor microbiome diversity has a powerful prognostic significance and can potentially modulate tumor sensitivity to gemcitabine. However, data from microbiome influence on PDAC outcome are still preliminary for establishing the microbiome as a solid biomarker. In addition, further studies have to be conducted to evaluate the role of fecal microbiota transplants as a possibility to increase chemotherapy sensitivity in PDAC patients.

## 8. Conclusions

Various new biomarkers for PDAC have emerged over the past decade, but none of these markers has been established in clinical routine. Our improved understanding of the tumor biology and microenvironment of PDAC increased the efforts on biomarker detection. Moreover, an increase in availability and reduction in cost for analytical methods, such as sequencing, supports a broad application of biomarker identification. However, several of these new approaches struggled to outperform CA 19-9, which is the only broadly used biomarker of PDAC for more than 20 years. A combination of markers or markers relying on panel or omics approaches to characterize disease-associated patterns, which overcome this limitation and implementation of new diagnostic, prognostic and predictive biomarkers for PDAC, could significantly improve the quality of care and ultimately survival of the pancreatic cancer patients.

## Figures and Tables

**Table 1:** Summary of diagnostic sensitivity and specificity of carbohydrate biomarkers for Pancreatic ductal adenocarcinoma (PDAC).

Biomarker, Serum	Reference	Sensitivity	Specificity	*n* of Patients
CA 19-9	[33]	81	90	1040 ^a^
	[28]	75.5	77.6	3497 ^a^
	[17]	79	82	2283 ^a^
	[34]	81	81	3125 ^a^
CEA	[35]	39	91	123
	[28]	39.5	81.3	3497 ^a^
	[32]	45	75	68
CA 125	[36]	66.8	83.3	110
	[35]	57	78	123
CA 50	[37]	84	85	200
	[29]	46.3	90.9	112
	[31]	82	78	129
CA 72-4	[31]	63.4	75.2	129
CA 242	[28]	67.8	83	3497 ^a^
	[31]	82	78	129
	[38]	60	76	68

^a^ meta-analysis.

**Table 2:** Plasma cell free DNA (cfDNA) and prognosis among patients with PDAC.

Year	Study Type	Stage	Patients	Marker	Measure	HR (95% CI) for Death	Ref
2019	Prospective cohort	Localized and LAPC	194	KRAS mutations	Presence of *KRAS*^mut^ cfDNA MAF 35%	2.8; (1.4–5.7) * 3.46 (1.4–8.5) *	[93]
2017	Retrospective cohort	Metastatic PDAC	188	KRAS codon 12 and ERBB2 mutations	Presence of *KRAS*^mut^/*ERBB2*^mut^ cfDNA	Associated with a trend towards shorter OS	[94]
2017	Prospective cohort	Advanced PDAC	27	KRAS MAF kinetics	KRAS MAF stability/decrease vs. increase	mOS 6.5 vs. 11.5 months (*p* = 0.009)	[95]
2015	Prospective cohort	Mixed localized and advanced PDAC	31	KRAS codon 12 mutations	Presence of *KRAS*^mut^ cfDNA is associated with shorter OS	12.2 (3.3–45.1) *	[91]
2016	Prospective cohort	Resectable PDAC	105	KRAS codon 12 mutations	Presence of *KRAS*^mut^ cfDNA	3.2 (1.8–5.4) *	[92]
2018	Prospective cohort	Mixed localized and advanced PDAC	106	KRAS codon 12/13 mutations	KRAS MAF > 0.415	1.73 (0.95–3.14) *	[88]
2018	Prospective cohort	Advanced PDAC	54	KRAS mutations	*KRAS*^mut^ concentration	Decrease in KRAS^mut^ correlates with response	[96]
2018	Prospective cohort	Advanced PDAC	61	Total cfDNA	cfDNA ≤ 167 bp above median plasma level	2.24 (1.09–4.59) *	[97]
2019	Prospective cohort	LAPC and metastatic PDAC	55	Copy numbers and KRAS mutations	CAN ^†^ KRAS copy number-*KRAS*^mut^ at baseline	7.09 (3.19–15.78) 10.94 (3.85–31.08) 3.46 (1.76–6.77)	[98]
2018	Retrospective cohort	Resectable PDAC	45	KRAS codon 12 mutations	Presence of post-operative *KRAS*^mut^ cfDNA	2.92 (1.11–5.62)	[99]
2018	Prospective cohort	Metastatic PDAC	17	KRAS mutations	Detectable vs. no detectable *KRAS*^mut^ cfDNA	mOS 8 v. 37.5 months (*p* < 0.004)	[100]
2017	Prospective cohort	Mixed localized and advanced PDAC	104	KRAS codon 12 mutations	Detectable vs. no detectable *KRAS*^mut^ cfDNA KRAS MAF tertiles	1.99 (1.13–3.5) * mOS 18.9 vs. 7.8 vs. 4.9 months (*p* < 0.001)	[101]
2015	Retrospective cohort	Mixed localized and advanced PDAC	127	KRAS codon 12 mutations and KRAS mutations concentrations	Detectable *KRAS*^mut^ *KRAS*^mut^ cfDNA concentration > 62 ng/mL	0.8 (0.48–1.3) ^††^ 2.8 (1.8–4.6)	[38]
2019	Prospective cohort	Metastatic PDAC	58	Custom NGS panel	MAF (per 1%) Detectable mutated cfDNA	1.05 (1.01–1.09) * 2.16 (1.21–3.85) ^††^	[89]
2016	Prospective cohort	Advanced PDAC	14	KRAS codon 12 mutations	Detectable *KRAS*^mut^	5.86 (*p* = 0.099)	[90]
2019	Prospective cohort	Advanced PDAC	38	Custom NGS panel	Pre-treatment/follow-up MAF	Not applicable	[102]

* multivariate COX analysis; ^†^ copy number alterations; ^††^ univariate Cox model; CNA, copy number alterations; MAF, mutant allele frequency; NGS, next-generation sequencing.
